# Supplementary material for: HIVprotI: an integrated web based platform for prediction and design of HIV proteins inhibitors
Source: J Cheminform. 2018 Mar 9;10:12. doi: 10.1186/s13321-018-0266-y (PMC5845081; doi:10.1186/s13321-018-0266-y)
Supplement: Supplementary file 2 — Additional file 2. Source code of HIVProtI web server. [file 13321_2018_266_MOESM2_ESM.zip › HIVprotI_Source-code/hivprotI/weka-3-6-6/doc/weka/gui/experiment/OutputFormatDialog.html]

OutputFormatDialog


---


|  |  |  |
| --- | --- | --- |
| **Overview** \| **Package** \| **Class** \| **Tree** \| **Deprecated** \| **Index** \| **Help** \| **Weka's home** | |  |
| **PREV CLASS**   **NEXT CLASS** | **FRAMES**    **NO FRAMES**     **All Classes** |  |
| SUMMARY: NESTED \| FIELD \| CONSTR \| METHOD | DETAIL: FIELD \| CONSTR \| METHOD |  |


---


## weka.gui.experiment Class OutputFormatDialog

```
java.lang.Object
  java.awt.Component
      java.awt.Container
          java.awt.Window
              java.awt.Dialog
                  javax.swing.JDialog
                      weka.gui.experiment.OutputFormatDialog
```

**All Implemented Interfaces:**: java.awt.image.ImageObserver, java.awt.MenuContainer, java.io.Serializable, javax.accessibility.Accessible, javax.swing.RootPaneContainer, javax.swing.WindowConstants

---

``` public class OutputFormatDialog extends javax.swing.JDialog ```

A dialog for setting various output format parameters.

**Version:**
:   $Revision: 7059 $

**Author:**
:   FracPete (fracpete at waikato dot ac dot nz)

**See Also:**: Serialized Form

---

**Nested Class Summary**

**Nested classes/interfaces inherited from class java.awt.Dialog**

`java.awt.Dialog.ModalExclusionType, java.awt.Dialog.ModalityType`

**Nested classes/interfaces inherited from class java.awt.Component**

`java.awt.Component.BaselineResizeBehavior`


| **Field Summary** | |
| --- | --- |
| `static int` | `APPROVE_OPTION`             Signifies an OK property selection. |
| `static int` | `CANCEL_OPTION`             Signifies a cancelled property selection. |

**Fields inherited from class java.awt.Dialog**

`DEFAULT_MODALITY_TYPE`

**Fields inherited from class java.awt.Component**

`BOTTOM_ALIGNMENT, CENTER_ALIGNMENT, LEFT_ALIGNMENT, RIGHT_ALIGNMENT, TOP_ALIGNMENT`

**Fields inherited from interface javax.swing.WindowConstants**

`DISPOSE_ON_CLOSE, DO_NOTHING_ON_CLOSE, EXIT_ON_CLOSE, HIDE_ON_CLOSE`

**Fields inherited from interface java.awt.image.ImageObserver**

`ABORT, ALLBITS, ERROR, FRAMEBITS, HEIGHT, PROPERTIES, SOMEBITS, WIDTH`


**Constructor Summary**

`OutputFormatDialog(java.awt.Frame parent)`
  
          initializes the dialog with the given parent frame.


| **Method Summary** | |
| --- | --- |
| `int` | `getMeanPrec()`             Gets the precision used for printing the mean. |
| `boolean` | `getRemoveFilterName()`             returns whether the filter classname is removed from the dataset name. |
| `int` | `getResult()`             the result from the last display of the dialog, the same is returned from `showDialog`. |
| `java.lang.Class` | `getResultMatrix()`             Gets the currently selected output format result matrix. |
| `boolean` | `getShowAverage()`             returns whether the average for each column is displayed. |
| `int` | `getStdDevPrec()`             Gets the precision used for printing the std. |
| `static void` | `main(java.lang.String[] args)`             for testing only. |
| `void` | `setMeanPrec(int precision)`             Sets the precision of the mean output. |
| `void` | `setRemoveFilterName(boolean remove)`             sets whether to remove the filter classname from the dataset name. |
| `void` | `setResultMatrix(java.lang.Class matrix)`             Sets the matrix to use as initial selected output format. |
| `void` | `setShowAverage(boolean show)`             sets whether the average for each column is displayed. |
| `void` | `setStdDevPrec(int precision)`             Sets the precision of the std. |
| `int` | `showDialog()`             Pops up the modal dialog and waits for cancel or a selection. |

**Methods inherited from class javax.swing.JDialog**

`getAccessibleContext, getContentPane, getDefaultCloseOperation, getGlassPane, getGraphics, getJMenuBar, getLayeredPane, getRootPane, getTransferHandler, isDefaultLookAndFeelDecorated, remove, repaint, setContentPane, setDefaultCloseOperation, setDefaultLookAndFeelDecorated, setGlassPane, setJMenuBar, setLayeredPane, setLayout, setTransferHandler, update`

**Methods inherited from class java.awt.Dialog**

`addNotify, getModalityType, getTitle, hide, isModal, isResizable, isUndecorated, setModal, setModalityType, setResizable, setTitle, setUndecorated, setVisible, show, toBack`

**Methods inherited from class java.awt.Window**

`addPropertyChangeListener, addPropertyChangeListener, addWindowFocusListener, addWindowListener, addWindowStateListener, applyResourceBundle, applyResourceBundle, createBufferStrategy, createBufferStrategy, dispose, getBufferStrategy, getFocusableWindowState, getFocusCycleRootAncestor, getFocusOwner, getFocusTraversalKeys, getGraphicsConfiguration, getIconImages, getInputContext, getListeners, getLocale, getModalExclusionType, getMostRecentFocusOwner, getOwnedWindows, getOwner, getOwnerlessWindows, getToolkit, getWarningString, getWindowFocusListeners, getWindowListeners, getWindows, getWindowStateListeners, isActive, isAlwaysOnTop, isAlwaysOnTopSupported, isFocusableWindow, isFocusCycleRoot, isFocused, isLocationByPlatform, isShowing, pack, paint, postEvent, removeNotify, removeWindowFocusListener, removeWindowListener, removeWindowStateListener, reshape, setAlwaysOnTop, setBounds, setBounds, setCursor, setFocusableWindowState, setFocusCycleRoot, setIconImage, setIconImages, setLocationByPlatform, setLocationRelativeTo, setMinimumSize, setModalExclusionType, setSize, setSize, toFront`

**Methods inherited from class java.awt.Container**

`add, add, add, add, add, addContainerListener, applyComponentOrientation, areFocusTraversalKeysSet, countComponents, deliverEvent, doLayout, findComponentAt, findComponentAt, getAlignmentX, getAlignmentY, getComponent, getComponentAt, getComponentAt, getComponentCount, getComponents, getComponentZOrder, getContainerListeners, getFocusTraversalPolicy, getInsets, getLayout, getMaximumSize, getMinimumSize, getMousePosition, getPreferredSize, insets, invalidate, isAncestorOf, isFocusCycleRoot, isFocusTraversalPolicyProvider, isFocusTraversalPolicySet, layout, list, list, locate, minimumSize, paintComponents, preferredSize, print, printComponents, remove, removeAll, removeContainerListener, setComponentZOrder, setFocusTraversalKeys, setFocusTraversalPolicy, setFocusTraversalPolicyProvider, setFont, transferFocusBackward, transferFocusDownCycle, validate`

**Methods inherited from class java.awt.Component**

`action, add, addComponentListener, addFocusListener, addHierarchyBoundsListener, addHierarchyListener, addInputMethodListener, addKeyListener, addMouseListener, addMouseMotionListener, addMouseWheelListener, bounds, checkImage, checkImage, contains, contains, createImage, createImage, createVolatileImage, createVolatileImage, disable, dispatchEvent, enable, enable, enableInputMethods, firePropertyChange, firePropertyChange, firePropertyChange, firePropertyChange, firePropertyChange, firePropertyChange, getBackground, getBaseline, getBaselineResizeBehavior, getBounds, getBounds, getColorModel, getComponentListeners, getComponentOrientation, getCursor, getDropTarget, getFocusListeners, getFocusTraversalKeysEnabled, getFont, getFontMetrics, getForeground, getHeight, getHierarchyBoundsListeners, getHierarchyListeners, getIgnoreRepaint, getInputMethodListeners, getInputMethodRequests, getKeyListeners, getLocation, getLocation, getLocationOnScreen, getMouseListeners, getMouseMotionListeners, getMousePosition, getMouseWheelListeners, getName, getParent, getPeer, getPropertyChangeListeners, getPropertyChangeListeners, getSize, getSize, getTreeLock, getWidth, getX, getY, gotFocus, handleEvent, hasFocus, imageUpdate, inside, isBackgroundSet, isCursorSet, isDisplayable, isDoubleBuffered, isEnabled, isFocusable, isFocusOwner, isFocusTraversable, isFontSet, isForegroundSet, isLightweight, isMaximumSizeSet, isMinimumSizeSet, isOpaque, isPreferredSizeSet, isValid, isVisible, keyDown, keyUp, list, list, list, location, lostFocus, mouseDown, mouseDrag, mouseEnter, mouseExit, mouseMove, mouseUp, move, nextFocus, paintAll, prepareImage, prepareImage, printAll, remove, removeComponentListener, removeFocusListener, removeHierarchyBoundsListener, removeHierarchyListener, removeInputMethodListener, removeKeyListener, removeMouseListener, removeMouseMotionListener, removeMouseWheelListener, removePropertyChangeListener, removePropertyChangeListener, repaint, repaint, repaint, requestFocus, requestFocusInWindow, resize, resize, setBackground, setComponentOrientation, setDropTarget, setEnabled, setFocusable, setFocusTraversalKeysEnabled, setForeground, setIgnoreRepaint, setLocale, setLocation, setLocation, setMaximumSize, setName, setPreferredSize, show, size, toString, transferFocus, transferFocusUpCycle`

**Methods inherited from class java.lang.Object**

`equals, getClass, hashCode, notify, notifyAll, wait, wait, wait`

**Field Detail**

### APPROVE\_OPTION

```
public static final int APPROVE_OPTION
```

:   Signifies an OK property selection.

    **See Also:**: Constant Field Values

---


### CANCEL\_OPTION

```
public static final int CANCEL_OPTION
```

:   Signifies a cancelled property selection.

    **See Also:**: Constant Field Values


**Constructor Detail**

### OutputFormatDialog

```
public OutputFormatDialog(java.awt.Frame parent)
```

:   initializes the dialog with the given parent frame.

    **Parameters:**: `parent` - the parent of this dialog


**Method Detail**

### setMeanPrec

```
public void setMeanPrec(int precision)
```

:   Sets the precision of the mean output.

    :   **Parameters:**: `precision` - the number of digits used in printing the mean

---


### getMeanPrec

```
public int getMeanPrec()
```

:   Gets the precision used for printing the mean.

    :   **Returns:**: the number of digits used in printing the mean

---


### setStdDevPrec

```
public void setStdDevPrec(int precision)
```

:   Sets the precision of the std. deviation output.

    :   **Parameters:**: `precision` - the number of digits used in printing the std. deviation

---


### getStdDevPrec

```
public int getStdDevPrec()
```

:   Gets the precision used for printing the std. deviation

    :   **Returns:**: the number of digits used in printing the std. deviation

---


### setResultMatrix

```
public void setResultMatrix(java.lang.Class matrix)
```

:   Sets the matrix to use as initial selected output format.

    :   **Parameters:**: `matrix` - the matrix to use as initial selected output format

---


### getResultMatrix

```
public java.lang.Class getResultMatrix()
```

:   Gets the currently selected output format result matrix.

    :   **Returns:**: the currently selected matrix to use as output

---


### setRemoveFilterName

```
public void setRemoveFilterName(boolean remove)
```

:   sets whether to remove the filter classname from the dataset name.

    :   **Parameters:**: `remove` - if true then the filter classname is removed

---


### getRemoveFilterName

```
public boolean getRemoveFilterName()
```

:   returns whether the filter classname is removed from the dataset name.

    :   **Returns:**: true if the filter classname is removed

---


### setShowAverage

```
public void setShowAverage(boolean show)
```

:   sets whether the average for each column is displayed.

    :   **Parameters:**: `show` - if true then an additional row with the average is printed.

---


### getShowAverage

```
public boolean getShowAverage()
```

:   returns whether the average for each column is displayed.

    :   **Returns:**: true if an additional row with the average is displayed

---


### getResult

```
public int getResult()
```

:   the result from the last display of the dialog, the same is returned
    from `showDialog`.

    :   **Returns:**: the result from the last display of the dialog; either APPROVE\_OPTION, or CANCEL\_OPTION **See Also:**: `showDialog()`

---


### showDialog

```
public int showDialog()
```

:   Pops up the modal dialog and waits for cancel or a selection.

    :   **Returns:**: either APPROVE\_OPTION, or CANCEL\_OPTION

---


### main

```
public static void main(java.lang.String[] args)
```

:   for testing only.

    :   **Parameters:**: `args` - ignored


---


|  |  |  |
| --- | --- | --- |
| **Overview** \| **Package** \| **Class** \| **Tree** \| **Deprecated** \| **Index** \| **Help** \| **Weka's home** | |  |
| **PREV CLASS**   **NEXT CLASS** | **FRAMES**    **NO FRAMES**     **All Classes** |  |
| SUMMARY: NESTED \| FIELD \| CONSTR \| METHOD | DETAIL: FIELD \| CONSTR \| METHOD |  |


---
